# Development and validation of cuproptosis-related genes in synovitis during osteoarthritis progress

**DOI:** 10.3389/fimmu.2023.1090596

**Published:** 2023-02-02

**Authors:** Bohan Chang, Zhehan Hu, Liang Chen, Zhuangzhuang Jin, Yue Yang

**Affiliations:** ^1^ Department of Rheumatology, The First Affiliated Hospital of China Medical University, Shenyang, Liaoning, China; ^2^ Department of Orthopedic Surgery, Shengjing Hospital of China Medical University, Shenyang, Liaoning, China; ^3^ Department of Emergence Medicine, Shengjing Hospital of China Medical University, Shenyang, Liaoning, China

**Keywords:** osteoarthritis, synovium, cuproptosis, immune infiltration, bioinformatic analysis, single-cell RNA-seq analysis

## Abstract

Osteoarthritis (OA) is one of the most common refractory degenerative joint diseases worldwide. Synovitis is believed to drive joint cartilage destruction during OA pathogenesis. Cuproptosis is a novel form of copper-induced cell death. However, few studies have examined the correlations between cuproptosis-related genes (CRGs), immune infiltration, and synovitis. Therefore, we analyzed CRGs in synovitis during OA. Microarray datasets (GSE55235, GSE55457, GSE12021, GSE82107 and GSE176308) were downloaded from the Gene Expression Omnibus database. Next, we conducted differential and subtype analyses of CRGs across synovitis. Immune infiltration and correlation analyses were performed to explore the association between CRGs and immune cell abundance in synovitis. Finally, single-cell RNA-seq profiling was performed using the GSE176308 dataset to investigate the expression of CRGs in the various cell clusters. We found that the expression of five CRGs (FDX1, LIPT1, PDHA1, PDHB, and CDKN2A) was significantly increased in the OA synovium. Moreover, abundant and various types of immune cells infiltrated the synovium during OA, which was correlated with the expression of CRGs. Additionally, single-cell RNA-seq profiling revealed that the cellular composition of the synovium was complex and that their proportions varied greatly as OA progressed. The expression of CRGs differed across various cell types in the OA synovium. The current study predicted that cuproptosis may be involved in the pathogenesis of synovitis. The five screened CRGs (FDX1, LIPT1, PDHA1, PDHB, and CDKN2A) could be explored as candidate biomarkers or therapeutic targets for OA synovitis.

## 1 Introduction

Osteoarthritis (OA) is one of the most common degenerative joint diseases in the older population, and the excruciation and disability resulting from it pose tremendous burdens on individuals and society (1). OA pathogenesis involves crosstalk between the synovium, cartilage, and subchondral bone (2). Recently, the goals of OA prevention and treatment have expanded to focus on the cartilage microenvironment. The synovium is one of the key constituents of the cartilage microenvironment (3, 4). Synovium serves as a two-way bridge for nutrients and the clearance of waste between the cartilage and bloodstream (5). Additionally, the OA synovium is characterized by lining hyperplasia, fibrosis, and stromal vascularization, which may contribute to the pain and swelling associated with OA (6). Therefore, the synovium is of great significance for joint homeostasis, and its pathogenesis is among the main drivers of joint cartilage destruction (7). However, there is still controversy regarding the mechanism of synovitis formation and its effect on cartilage during the OA process.

Copper is an indispensable trace element and serves as a co-factor for enzymes in physical conditions were inappropriate concentrations may be toxic to cells (8, 9). Notably, the metabolic disorder caused by copper correlates strongly with OA pathogenesis (10). Copper deficiency could be detrimental to cartilage integrity and increase the prevalence of OA, which may arise due to issues in the activity of lysine oxidase and impaired cross-linking between collagen and elastin (11, 12). In contrast, epidemiological studies have pointed out that copper overload is closely associated with a high risk of OA. However, the mechanism is poorly understood (10, 13). Cuproptosis is copper-dependent and different from necrosis, pyroptosis, and other types of cell death (9, 14). As a novel cell death, cuproptosis has attracted great attention. It occurs *via* the direct binding of excess copper to the lipoylated components of the tricarboxylic acid (TCA) cycle when the mitochondrial respiration chain is disrupted (9). Furthermore, cell death is closely associated with OA synovitis, such as in fibroblasts and monocytes (15, 16). Therefore, it is reasonable to speculate that cuproptosis may be involved in the development of OA synovitis. Cuproptosis-related genes show promise as novel targets for OA treatment.

Currently, bioinformatics technology is increasingly being used to analyze molecular mechanisms and network relationships for OA prevention, diagnosis, and progression monitoring (17). The synovium contains immune cells, including macrophages, neutrophils, and other immune cells, whose interactions are thought to drive the enzymatic activity of cartilage to aggravate the feed-forward loop of degradation (18). Using a computational analytical tool, such as CIBERSORT, is an efficient way to estimate immune cell abundance in a mixed cell population based on microarray gene expression data (19). With advances in single-cell RNA-seq profiling, disease-associated cell clusters in human tissues, such as the synovium, may be identified at high resolution in an unbiased manner (15). However, few studies have used bioinformatic techniques to explore cuproptosis-related genes (CRGs) and cell clusters in OA synovitis, which may hail a new avenue for further research on OA.

Therefore, we designed this study to identify and elucidate the function of CRGs in synovitis as OA progresses. First, we obtained microarray datasets GSE55235 and GSE55457 as internal datasets to determine the expression distribution of CRGs, followed by an analysis of functional relevance, including subgroup identification and GSEA. Second, we explored the abundance of 22 immune cells and their correlation with the expression of CRGs, based on an internal dataset. Third, we validated the expression of CRGs in external datasets (GSE12021 and GSE82107) and with single-cell RNA-seq profiling (GSE176308). This study is the first to identify five differentially expressed CRGs (FDX1, LIPT1, PDHA1, PDHB, and CDKN2A) in OA synovitis, offering a novel direction for exploring the pathogenesis and treatment of OA.

## 2 Materials and methods

### 2.1 Microarray data processing and analysis of cuproptosis-associated genes

We downloaded transcriptomic and clinical microarray information (GSE55235, GSE55457, GSE12021, GSE82107, and GSE176308) from the GEO website. The experiments for microarray datasets GSE55235, GSE55457, and GSE12021 were conducted on the GPL96 platform [(HG-U133A) Affymetrix Human Genome U133A Array], whereas that for GSE82107 was conducted on the GPL570 platform [(HG-U133_Plus_2) Affymetrix Human Genome U133 Plus 2.0 array]. GSE176308 was used for single-cell RNA-seq profiling. Joint synovial samples from GSE55235 and GSE55457 were included to screen differentially expressed genes (DEGs) and analyze the expression distribution of CRGs. GSE12021 and GSE82107 were used to validate the CRGs. Gene expression profiling data were subjected to log2 transformation for normalization. The R package limma (version 3.40.2) was used to analyze differential expression levels. Genes with an adjusted *P*-value < 0.05 and |log_2_ fold change (FC)| > 2 were defined as DEGs. The removeBatchEffect function of the limma package in the R software was used to eliminate batch effects. A boxplot was used to illustrate the preprocessing results of the microarray, and principal component analysis (PCA) was performed using the ggord package in the R software. A heatmap package was used to draw the heatmap to illustrate the top 50 DEGs. The ClusterProfiler package (version 3.18.0) of R was used for the Gene Ontology (GO) function and Kyoto Encyclopedia of Genes and Genomes (KEGG) of potential targets. The expression of the CRGs is displayed in the form of boxplots with scatter points.

### 2.2 Subtyping of microarrays

Briefly, the consistency analysis was performed using the R package ConsensusClusterPlus (v1.54.0), where the maximum number of clusters was 6 and 80% of the total samples were extracted 100 times, clusterAlg = “hc”, innerLinkage = ‘ward.D2’. Clustering heatmaps were analyzed using the R software package pheatmap (v1.0.12). The gene expression heatmap retained genes with a variance greater than 0.1. If the number of input target genes was more than 1000, they were extracted and sorted according to the variance from large to small to display the top 25% of genes.

### 2.3 Protein-protein interaction (PPI) network construction

The functional interactions of the DEGs were annotated using the STRING online database (http://string-db.org). A combined score of more than 0.4 was considered as the inclusion criterion, and disconnected points were not displayed. The Cytoscape software (version 3.9.1) was used to visualize the PPI network. A node in the PPI network was considered to be the protein product of the gene. The top 50 hub genes were selected based on the molecular complex detection (MCODE) algorithm in Cytoscape.

### 2.4 Gene set enrichment analysis (GSEA)

Pathway enrichment analysis was performed using GSEA. Briefly, GSEA software (version 3.0) was obtained from http://software.broadinstitute.org/gsea/index.jsp. The samples of microarrays GSE55235 and GSE55457 were divided into a high-expression group (≥ 50%) and a low-expression group (< 50%) based on the expression of target genes. The c2.cp.kegg.v7.4.symbols.gmt subset was downloaded from the Molecular Signatures Database (http://www.gsea-msigdb.org/gsea/downloads.jsp) to assess the relevant pathways and molecular mechanisms. In the GSEA runs, the minimum gene set size was set to 5, and the maximum gene set size was set to 5000. *P* < 0.05 and false discovery rate (FDR) *P*-value < 0.25 were considered statistically significant.

### 2.5 Immune cell landscape and matrix correlation analysis

CIBERSORT, a deconvolution algorithm, was used to estimate the immune cell abundance from normalized microarrays (https://cibersort.stanford.edu/). The immune cell types were as follows: neutrophils, eosinophils, activated mast cells, resting mast cells, activated dendritic cells, resting dendritic cells, M0 macrophages, M1 macrophages, M2 macrophages, monocytes, activated NK cells, resting NK cells, γδ T cells, regulatory T cells, helper T cells, naïve CD4^+^ T cells, memory-activated CD4^+^ T cells, memory-resting CD4^+ ^T cells, CD8^+^ T cells, plasma cells, memory B cells, and naïve B cells. Permutations were set to 1000, and quantile normalization was used for GSE55235 and GSE55457. Furthermore, the correlation between immune cell abundance and CRGs was investigated using the web-based tool Sanger Box (http://vip.sangerbox.com/home.html), which provides a matrix visualization and analysis platform designed to support visual data exploration.

### 2.6 Data quality control for single-cell RNA-seq analysis

Briefly, the R package Seurat (version 2.2.0) was used to filter out cells of poor quality in GSE176308 during data preprocessing. We set a threshold for the number of RNAs and the percentage of mitochondrial RNAs in a single cell, such that the minimum amount of RNAs was 200, the maximum amount of RNAs was 6000, and the percentage of mitochondrial RNAs was no more than 10%. Several situations could be excluded during this process, including low-quality cells, empty droplets, double or multiple cells with single droplets, and mitochondrial RNA contamination. Furthermore, we excluded genes detected in less than three cells and cells with less than 200 genes.

### 2.7 Identification of cell clusters

The corrected, normalized data metric was applied to the standard analysis described in the Seurat R package. Variable genes were extracted for PCA, which was visualized and clustered using the UMAP method. Cell clustering was performed using the FindClusters function (resolution = 0.5) in the Seurat R package. Subpopulations were named according to TOP10 marker genes and annotated using different colors.

### 2.8 Expression analysis in single-cell RNA-seq analysis

With the help of online tools (https://www.home-for-researchers.com), we observed the expression of CRGs across different samples and cell populations. The AverageExpression function was used to calculate the average expression value of genes in various cell clusters or different groupings.

### 2.9 Statistical analysis

The Shapiro-Wilk test was used to verify the normality of the data, and Levene’s test was used to assess the homogeneity of variance. The Student’s t-test was used to evaluate the differences in normally distributed data with homogeneity of variance. The Wilcoxon rank-sum test was used when the normality or homogeneity of variance was not satisfied. Statistical analyses were conducted using SPSS version 21.0 (IBM Corp., Armonk, NY, USA). Statistical significance was set at *P* < 0.05.

## 3 Results

### 3.1 Bioinformatic characterization of DEGs in internal datasets

**Figure 1:**
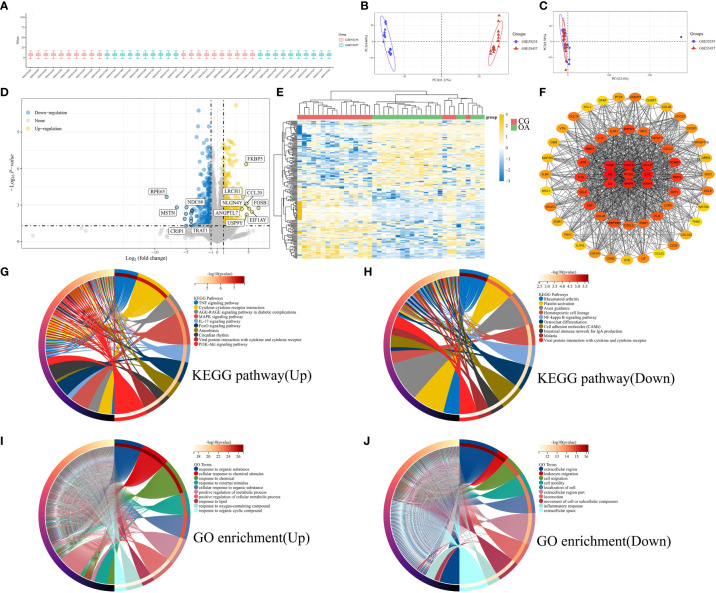
Bioinformatic characterization of DEGs in internal datasets. **(A)** Boxplot of normalized microarray data. **(B)** Visualization of PCA results before batch effect removal. **(C)** Visualization of PCA results after batch effect removal. **(D)** Volcano plot of DEGs. **(E)** Heatmap of the TOP50 DEGs. **(F)** PPI network of top 60 hub genes. **(G)** KEGG pathway of upregulated genes. **(H)** KEGG pathway of downregulated genes. **(I)** GO enrichment of upregulated genes. **(J)** GO enrichment of downregulated genes. PCA, principal component analysis; DEGs, differentially expressed genes; PPI, protein-protein interaction; KEGG, Kyoto Encyclopedia of Genes and Genomes; GO, Gene Ontology function.

We combined microarray data from datasets GSE55235 and GSE55457 to be used as the internal datasets, using 10 normal and 10 OA samples from each dataset. Box plots represent the distribution of differences, indicating that the internal datasets were normalized (**Figure 1A**). The PCA results suggested that the internal datasets had good repeatability after removing the batch effect (**Figures 1B, C**). An adjusted *P*-value < 0.05 and |log_2_(FC)| > 2 were used as the cut-off thresholds. A total of 317 upregulated and 276 downregulated genes were identified as DEGs from the internal datasets. A volcano plot was created based on the threshold value of the fold change (**Figure 1D**). The expression levels of the top 50 upregulated and downregulated DEGs are displayed in a heatmap (**Figure 1E**). The yellow color indicates upregulated genes, and the blue represents downregulated genes. We constructed a PPI network of the identified DEGs using the STRING database. The genes with top 60 scores were screened and visualized as hub genes using the MCC mode in Cytoscape software (**Figure 1F**). The result of KEGG pathways are displayed in **Figures 1G** (upregulated) and **H** (downregulated), which were mainly enriched in the PI3K-Akt and MAPK signaling pathways, cytokine-cytokine receptor interaction, and axon guidance. The GO enrichment results are illustrated in **Figures 1I** (upregulated) and **J** (downregulated), suggesting that DEGs were mainly enriched in the cellular response to chemical stimuli and organic substances and positive regulation of metabolic processes.

### 3.2 Identification of CRGs in internal microarray datasets

**Figure 2:**
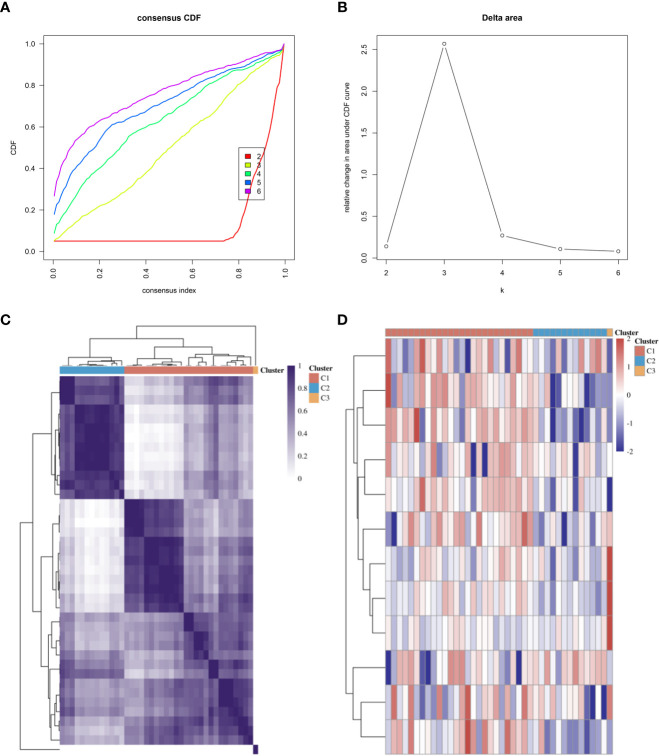
Subtyping of microarrays based on CRGs. **(A)** CDF curves. **(B)** Delta area of CDF curves. **(C)** Consistency of clustering results heatmap at K = 3. **(D)** The expression heatmap of CRGs in different subgroups; red represents high expression, and blue represents low expression. CDF, cumulative distribution function; CRGs, cuproptosis-related genes.

We referred to previous studies and enrolled several genes as CRGs, including FDX1, LIAS, DLD, LIPT1, DLAT, PDHA1, PDHB, MTF1, GLS, CDKN2A, SLC31A1, and ATP7B (9, 20, 21). Consensus clustering analysis was performed to identify subgroups according to CRG expression. The cumulative distribution function (CDF) curve needed to be stable, and the delta area curve provided an inflection point (**Figures 2A, B**). Ultimately, we determined that 3 was the optimal number of subgroups. The consistency of the heatmap of the clustering results at K = 3 is shown in **Figure 2C**. In the expression heatmap of CRGs in different subgroups, red represents high expression, and blue reflects low expression (**Figure 2D**).

**Figure 3:**
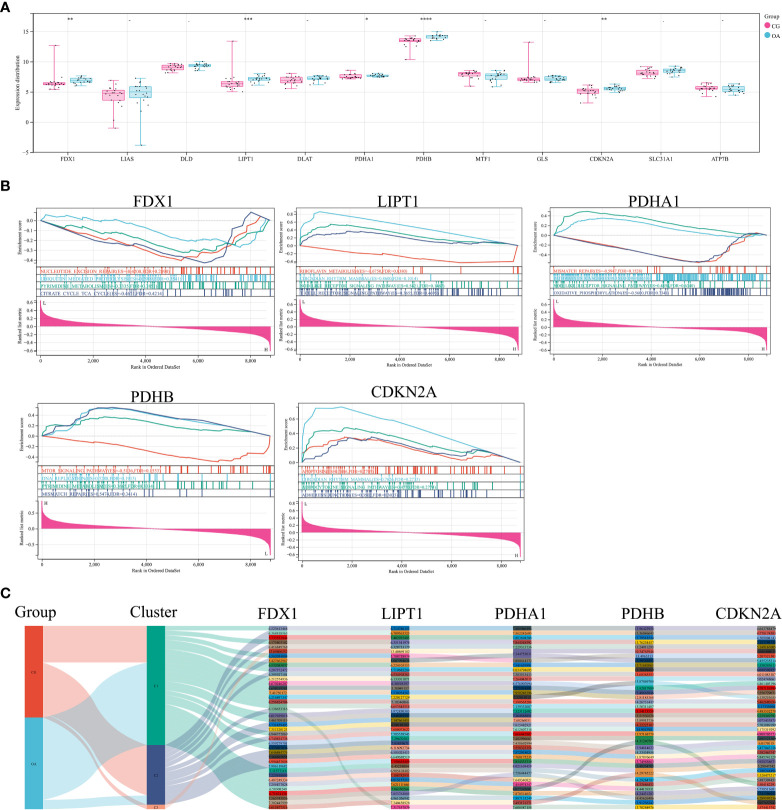
CRGs in internal datasets. **(A)** Expression distribution of CRGs. **(B)** GSEA of differentially expressed CRGs. **(C)** Sankey diagram of differentially expressed CRGs. CRGs, cuproptosis-related genes; GSEA, gene set enrichment analysis; CG, control group; OA, osteoarthritis group. ^*^ *P* < 0.05 ^**^ *P* < 0.01, ^***^ *P* < 0.001, ^****^ *P* < 0.0001 *versus* CG.

The expression distribution of CRGs in internal datasets is illustrated in **Figure 3A**. FDX1, LIPT1, PDHB, and CDKN2A were significantly upregulated in OA samples (**Figure 3A**, ^**^ *P* < 0.01, ^***^ *P* < 0.001, ^****^ *P* < 0.0001). The difference in PDHA1 expression was minor but statistically significant (**Figure 3A**, ^*^ *P* < 0.05). GSEA was used to elucidate the biological pathways associated with the expression of these five CRGs in the OA synovium (**Figure 3B**). The results included the citrate cycle, nod-like receptor signaling pathway, and mismatch repair. The Sankey diagram illustrates the original data of these five CRGs in each sample of the internal dataset (**Figure 3C**).

### 3.3 Association between CRGs and immune cell infiltration

**Figure 4:**
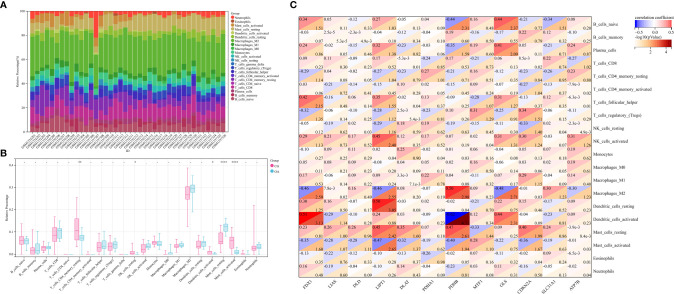
Profiles of immune cell subtype distribution patterns in GSE55235 and GSE55457 cohorts. **(A)** Visualization of the relative percentage of 22 immune cell types in each sample. **(B)** Boxplot of the abundance of the 22 immune cell types. **(C)** Matrix correlation between CRGs and immune cell abundance. CRGs, cuproptosis-related genes; CG, control group; OA, osteoarthritis group. *P < 0.05, **P < 0.01, ****P < 0.0001 versus CG.

Stacked histograms show the abundance distribution of 22 immune cell types in each sample (**Figure 4A**). Different colors represent different types of immune cells. The height of each color represents the percentage of cells in the sample, and the sum of the percentages was 1. Interestingly, the main infiltrating cells included resting and activated mast cells, M2 macrophages, monocytes, follicular helper T cells, regulatory T cells, resting memory CD4^+^ T cells, CD8^+^ T cells, and naïve B cells. Compared with CG synovium, the OA synovium generally contained a higher proportion of resting mast cells, whereas resting memory CD4^+^ T cells, resting NK cells, activated dendritic cells, and activated mast cell fractions were relatively lower (**Figure 4B**). The proportion of CRG expression and the abundance of immune cells were correlated from weak to moderate. For instance, the correlation between PDHB and activated dendritic cells was −0.62, and the correlation between FDX1 and activated dendritic cells was 0.51, among others (**Figure 4C**). Additionally, naïve and γδ T cells were not detected in the internal datasets. Therefore, the matrix correlation analysis could not be performed on these two cell populations.

### 3.4 Validation of CRGs in external datasets

**Figure 5:**
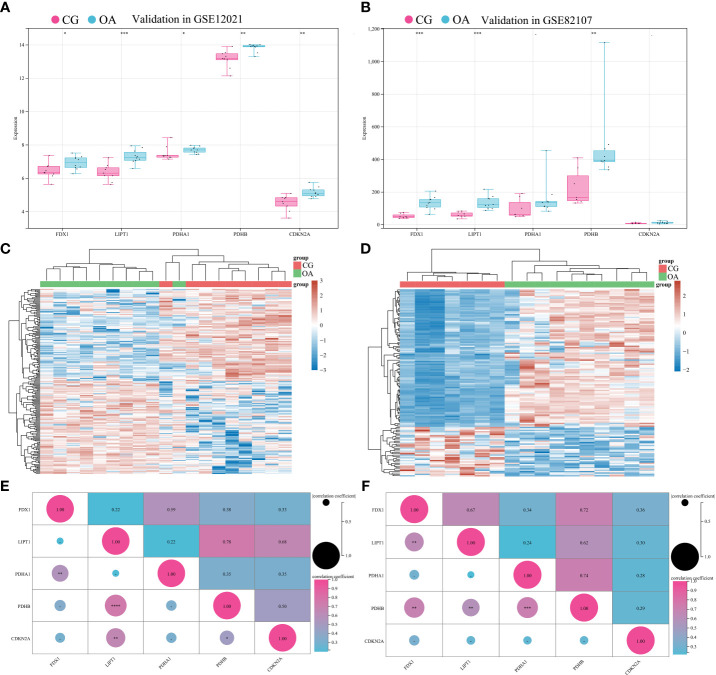
Validation of differentially expressed CRGs in external datasets. **(A)** Expression distribution in GSE12021. **(B)** Expression distribution in GSE82107. **(C)** Heatmap of GSE12021. **(D)** Heatmap of GSE82107. **(E)** Matrix correlation of differentially expressed CRGs in GSE12021. **(F)** Matrix correlation of differentially expressed CRGs in GSE82107. CRGs, cuproptosis-related genes; CG, control group; OA, osteoarthritis group. *P < 0.05, **P < 0.01, ***P < 0.001, ****P < 0.0001 versus CG.

To validate the associations between differentially expressed CRGs and the OA synovium, GSE12021 and GSE82107 were collected for independent validation. GSE12021 enrolled 9 samples in CG and 10 samples in OA on the GPL96 platform, while GSE82107 included 7 samples in CG and 10 samples in OA on the GPL570 platform. The standardization of external datasets is shown in **Supplemenatry Figure 1**. The expression of FDX1, LIPT1, and PDHB significantly increased in both GSE12021 and GSE82107 (**Figures 5A, B**, ^*^ *P* < 0.05, ^**^ *P* < 0.01, ^***^ *P* < 0.001). The expression of PDHA1 and CDKN2A was significantly increased in GSE12021 but not in GSE82107 (**Figure 5A**, ^*^ *P* < 0.05, ^**^ *P* < 0.01). **Figures 5C, D** illustrate the expression heatmaps of the top 50 upregulated and downregulated genes in GSE12021 and GSE82107, respectively. **Figures 5E, F** show the matrix correlation of these differentially expressed CRGs based on GSE12021 and GSE82107, respectively. A moderate correlation was observed between the expression of these five CRGs.

### 3.5 Expression prediction of CRGs in single-cell RNA-seq profiling

**Figure 6:**
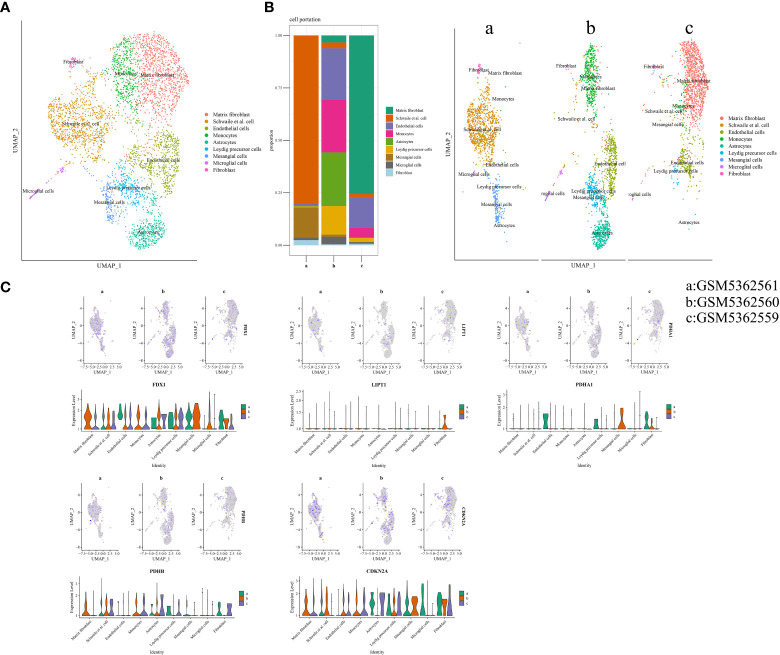
Cuproptosis in single-cell RNA-seq profiling. **(A)** Annotated cell population map. **(B)** The proportion of each cell’s cluster. **(C)** Expression distribution of differentially expressed CRGs in single-cell RNA-seq profiling. CRGs, cuproptosis-related genes.

Three samples (GSM5362559, GSM5362560, and GSM5362561) were obtained from the GSE176308 dataset for single-cell RNA-seq profiling. The synovial sources of GSM5362559, GSM5362560, and GSM5362561 were painful areas in end-stage OA, painful areas in early-stage OA, and non-painful areas in early-stage OA, respectively. Before the identification of cell clusters, the cells needed quality control, which included the number of featured RNA, number of RNA counts, percentage of mitochondrial RNA, removal of batching effect, and screening of highly variable feature genes. The quality control information is shown in **Supplementary Figure 2**. Cell clusters are shown in **Figure 6A**, containing matrix fibroblasts, Schwalie cells, endothelial cells, monocytes, astrocytes, Leydig precursor cells, mesangial cells, microglial cells, and fibroblasts. As shown in **Figure 6B**, the proportions of the different cell populations varied significantly. For example, fibroblasts gradually iterate into matrix fibroblasts as OA progresses. **Figure 6C** illustrates the expression of FDX1, LIPT1, PDHA1, PDHB, and CDKN2A in different samples and cell populations and is displayed in the form of a violin diagram.

## 4 Discussion

OA is one of the most common form of arthritis and is primarily responsible for pain and disability in the older population worldwide (22). During OA process, the synovium in the articular cavity is influenced by long-term stimulation to cause villous hyperplasia and erosion of articular cartilage, which aggravates the pathogenesis and symptoms of OA (23). Synovitis pathogenesis is triggered by a combination of multiple factors and genes through interactions and network regulation. In this study, we first identified 5 differentially expressed CRGs (FDX1, LIPT1, PDHA1, PDHB, and CDKN2A) as key genes in synovial cuproptosis during OA progression using bioinformatic analysis. Second, we observed the infiltration of abundant and various types of immune cells into the synovium during OA, and the abundance of several immune cells was correlated with the expression of CRGs. Third, the single-cell RNA-seq profile elucidated the change in the proportion of cell clusters in the synovium and the expression distribution of CRGs in the corresponding cell clusters during OA. These findings reveal a path for future exploration of the pathological mechanisms and therapeutic targets for OA.

Intracellular copper homeostasis is critical for cellular physiology and survival (24). Copper functions as a catalytic cofactor and drives many physiological processes, such as mitochondrial respiration, antioxidant defense, and bio-compound synthesis (25). The process by which copper induces cell death by targeting lipoylated TCA cycle proteins is called cuproptosis (9). Accumulated copper impairs the function of enzymes in the TCA cycle *via* lipoylation modification (26). The toxicity of copper has also been linked to the disruption of iron-sulfur (Fe-S) cluster formation, leading to cell death (27). TCA cycle disturbance and elevated glycolysis have also been reported in the OA synovium (28). Furthermore, copper levels were significantly increased in samples from OA patients (13). Mendelian randomization analyses has confirmed that a genetic predisposition to high copper status can increase the chance of OA (10). These findings offer a solid basis for exploring the role of cuproptosis in OA synovitis.

We explored the expression distribution of 12 CRGs. Seven cuproptosis-activated genes (FDX1, LIAS, LIPT1, DLD, DLAT, PDHA1, and PDHB) and three cuproptosis-suppressed genes (MTF1, GLS, and CDKN2A) were obtained from a previous study that first identified cuproptosis (9). Given the transport of copper, this study also included two genes encoding copper transporter proteins: ATP7B and SLC31A1 (20, 21). Five differentially expressed CRGs (FDX1, LIPT1, PDHA1, PDHB, and CDKN2A) were screened. FDX1 and LIPT1 are involved in the lipoic acid pathway, which is an essential step in lipid acylation, and lipoylated TCA cycle proteins are necessary targets for cuproptosis (9). FDX1 (Ferredoxin 1) is thought to be an upstream regulator of protein lipoylation and has responsiveness to copper ionophore (9). A recent study indicated that copper ionophore reduced an FDX1-dependent Fe-S cluster biogeneis (24). LIPT1 (lipoyltransferase-1) transfers lipoic acid from the H protein of the glycine cleavage system to the E2 subunit of 2-ketoacid dehydrogenase, and is involved in lipoic acid regulation LIPT1 deficiency which was demonstrated to suppress TCA cycle (29, 30). In addition, PDHA1 (pyruvate dehydrogenase E1 component subunit alpha), PDHB (pyruvate dehydrogenase B), and CDKN2A (cyclin-dependent kinase inhibitor 2A) are involved in the formation of PDHC (pyruvate dehydrogenase complex) and may unlink glycolysis and the TCA cycle during cuproptosis (31–33). We predicted that the expression of these five genes may increase in the OA synovium, which could be associated with copper overload or cuproptosis. Our prediction aligns with previous research on the expression of CRGs during cuproptosis (9). Therefore, the regulation of these five genes (FDX1, LIPT1, PDHA1, PDHB, and CDKN2A) on cuproptosis deserves to be explored in future studies on OA synovitis.

Another point of concern is the immune infiltration of the synovium. Many distinct infiltration patterns in the synovium have recently been discovered, such as the polarization of CD4^+^ T cells toward activated Th1 cells and the polarization of M1 macrophages toward M2 macrophages (19, 34). We explored the correlation between cuproptosis and immune cell abundance to identify the decrease in components of immune cells, which will be beneficial for developing new immunotherapeutic targets of OA. Based on our findings, macrophages and mast cells are worthy of discussion. Macrophages are much more abundant than other types of immune cells in synovial tissue. An imbalance in the ratio of the M1 and M2 subtypes exacerbates synovitis progression (35). The increased number of M1 macrophages and the high proportion of M2 macrophages have clinical diagnostic value for OA synovitis (36). Both M1 and M2 subtype macrophages can coexist in the synovium during OA, which makes it difficult to distinguish the polarization state of synovial macrophages (37). However, the effects of macrophages during OA are unassailable. In addition, mast cells were significantly silenced in the OA synovium compared to those in the normal synovium. Relatively high infiltration of mast cells was observed in OA synovium and could have been responsible for increased concentrations of mast cell mediators in synovial fluid, such as histamine and tryptase (37, 38). Many mediators of mast cells can suppress osteoblast activity or promote osteoclastogenesis, deteriorating the imbalanced bone-cartilage metabolism during the OA process (37, 39). The above literature evidence, together with our bioinformatic results, indicate that resting mast cells may be related to structural damage in OA, and this should be addressed in future studies. Furthermore, we explored the correlation between CRG expression and immune cell abundance. FDX1, LIPT1, and PDHB were associated with an abundance of macrophages and mast cells. These data must be interpreted with caution because the results of immune infiltration were predicted based on resources from public databases rather than experiments. Further experimental verification is required to confirm this correlation.

Single-cell RNA-seq profiling provides an opportunity to analyze tissues with multicellular components. Synovial fibroblasts are the major constituents responsible for the pathogenesis of synovitis (40). The maintenance of synovitis may be related to the synovial blood supply and the heterogeneity of resident monocytes (41). The synovium has nerve tissue that makes it sensitive to mechanical stimulation and can precisely localize pain (42). Increased endothelial cells and monocytes protect the synovium with proper blood supply and immune response during the early stages of OA (43). Synovial fibroblasts greatly reduce as OA progresses and can be decompensated by matrix fibroblasts. However, matrix fibroblasts may not allow the synovium to perform its original function. Surprisingly, the trends of FDX1, LIPT1, PDHA1, PDHB, and CDKN2A varied in different cell populations. Thus, different cell clusters may need to be analyzed in future studies on the synovium.

This study had several limitations. First, the findings are based on retrospective data obtained from public databases. Additional real-world sequencing data are required to improve the predictive power of these results. Secondly, the underlying regulatory and downstream mechanisms of CRGs in OA require further investigation. Third, the number of cases of cuproptosis is currently limited, and more potential genes in the initial stages or regulation of cuproptosis need to be explored.

## 5 Conclusions

Our study is the first to integrate microarray samples with single-cell RNA-seq profiles to investigate the expression of CRGs in OA synovitis while exploring the correlation of their expression with immune infiltration. We demonstrated that five screened CRGs (FDX1, LIPT1, PDHA1, PDHB, and CDKN2A) were significantly elevated in the OA synovium compared to healthy controls. Furthermore, the abundance of several immune cells in the synovium correlated with the expression of CRGs. In addition, single-cell RNA-seq profiles revealed that the proportion of cell clusters in the synovium changed during OA and elucidated the expression distribution of CRGs in the corresponding cell clusters. Hence, the five screened CRGs may be novel candidate biomarkers or therapeutic targets for OA research in the future.

## Data availability statement

The datasets GSE55235, GSE55457, GSE12021, GSE82107, GSE176308 for this study can be found in the GEO database (https://www.ncbi.nlm.nih.gov/geo/).

## Author contributions

BC: Conceptualization, methodology, data analysis, writing the original draft, reviewing, and editing. ZH and LC: Review and editing. ZJ and YY: Conceptualization, methodology, data analysis, review, editing, and funding acquisition. All authors contributed to the article and approved the submitted version.
