# Supplementary figures and images for: Development and validation of cuproptosis-related genes in synovitis during osteoarthritis progress
Source: Front Immunol. 2023 Feb 2;14:1090596. doi: 10.3389/fimmu.2023.1090596 (PMC9932029; doi:10.3389/fimmu.2023.1090596)

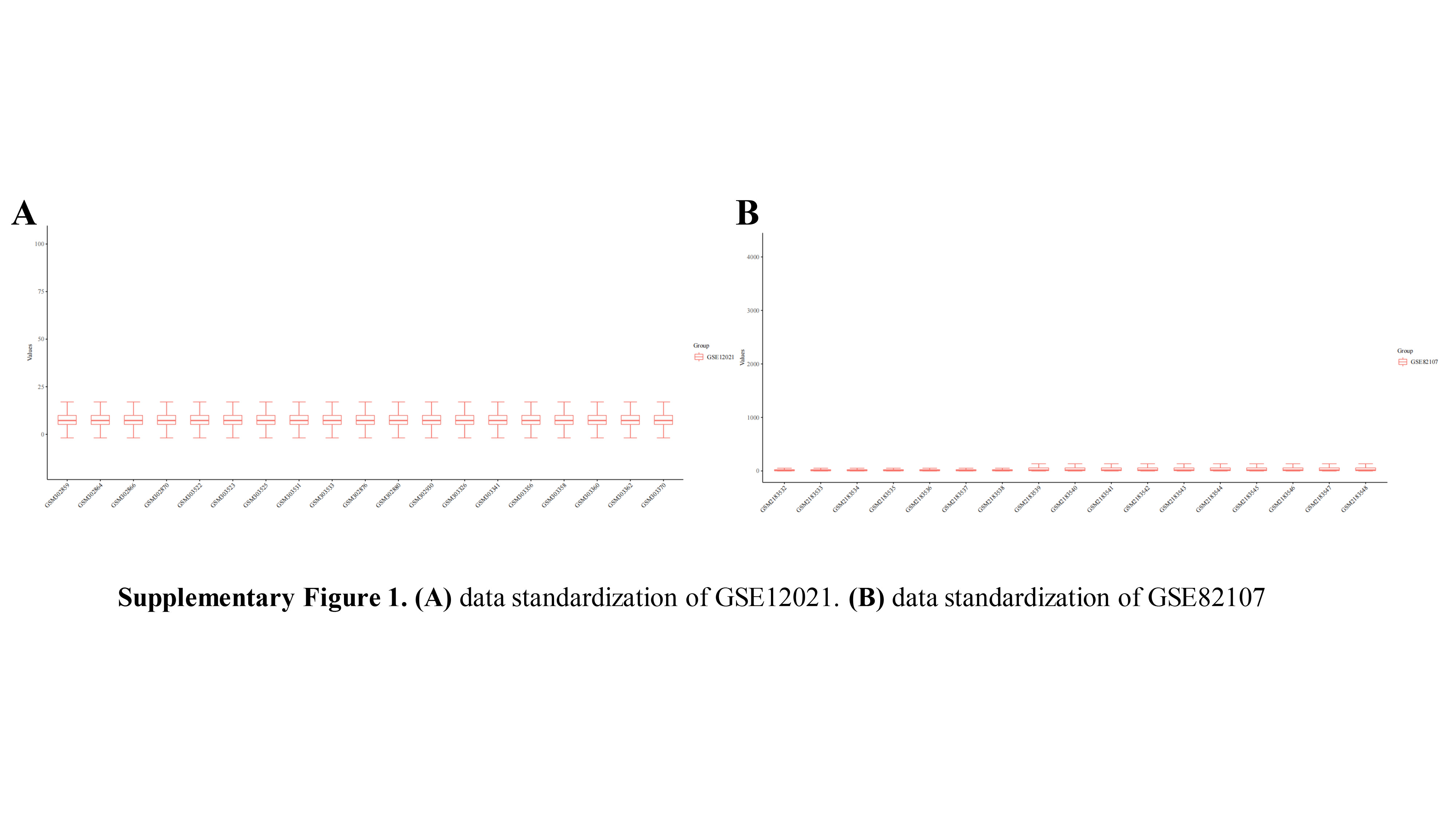

Supplement: Supplementary file 1 [file Image_1.tif]

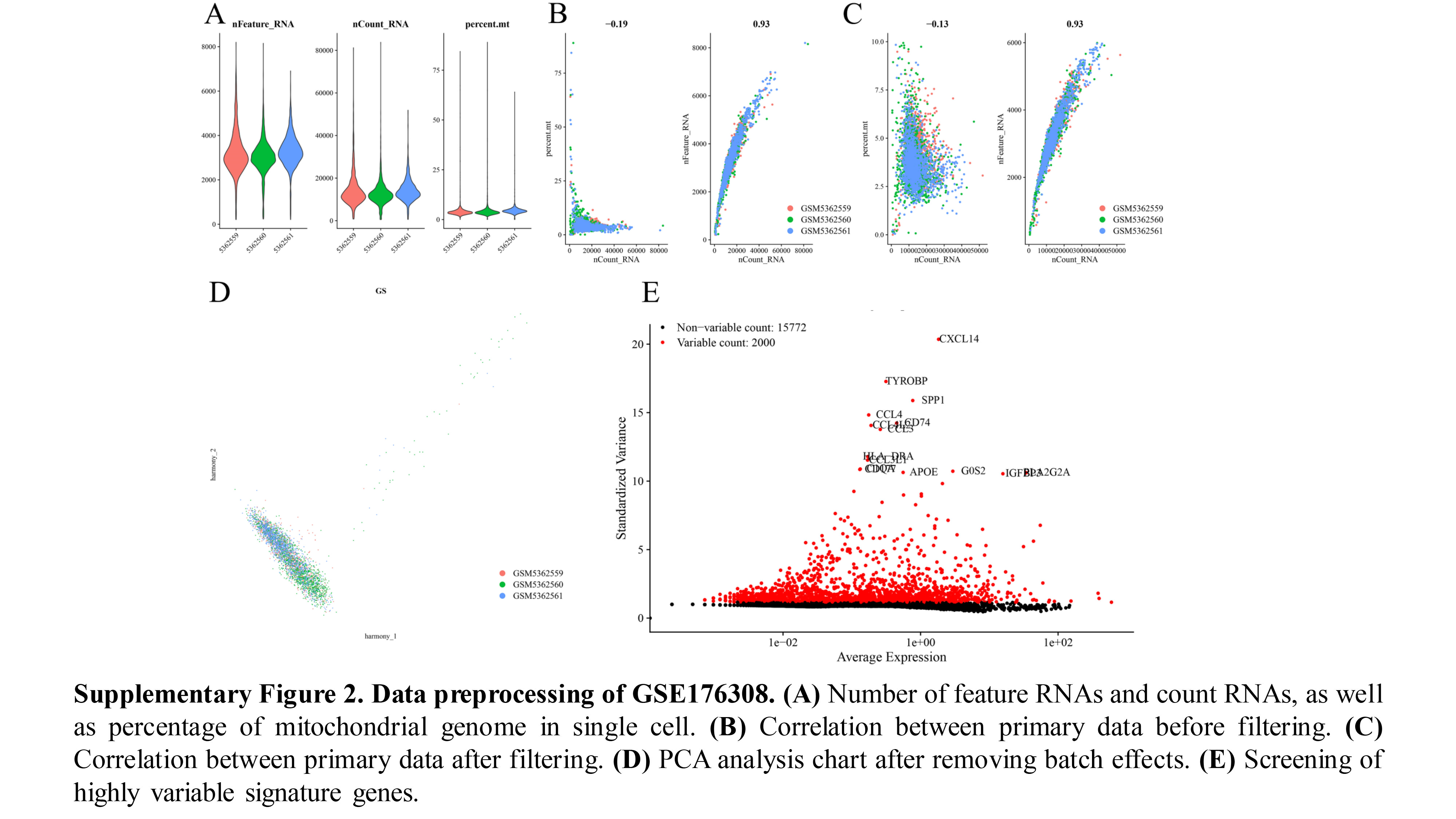

Supplement: Supplementary file 2 [file Image_2.tif]
